# Syrup of Valerian

**Published:** 1842-05-14

**Authors:** 


*Syrup of Valerian.*—Messrs. Procter and Turnpenny offer the following formulæ:—

1st. Take of Valerian, in powder, ℥j. Water, ℥iij. Sugar, in coarse powder, ℥vj.

Macerate the Valerian in the water for forty-eight hours, and displace so as to obtain three fluid ounces of infusion; then, having placed the sugar in a displacement funnel, treat it with the infusion until it is all dissolved.

2d. Take of Valerian, in powder, ℥j. Water, ℥ijss. Alcohol, ℥j. Sugar, ʒvj.

Proceed precisely as in the preceding formula.

There are some occasions where the effect of Valerian is wanted, unassociated with that produced by the presence of alcohol. The first formula embraces this idea, and fully answers its object. The second, where alcohol is no objection, forms a more ready means of exhausting the Valerian.

